# TRIM8 promotes ovarian cancer proliferation and migration by targeting VDAC2 for ubiquitination and degradation

**DOI:** 10.1002/cam4.7396

**Published:** 2024-06-17

**Authors:** Fei Wu, Jiaqi Xu, Xin Jin, Yue Zhu, Wenxin Gao, Meng Liu, Yan Zhang, Weifeng Qian, Xiaoyan Huang, Dan Zhao, Guannan Feng, Shunyu Hou, Xiaoxue Xi

**Affiliations:** ^1^ Department of Obstetrics and Gynecology The Affiliated Suzhou Hospital of Nanjing Medical University, Gusu School, Nanjing Medical University, Suzhou Municipal Hospital Suzhou Jiangsu China; ^2^ Department of Breast and Thyroid Surgery The Affiliated Suzhou Hospital of Nanjing Medical University, Gusu School, Nanjing Medical University, Suzhou Municipal Hospital Suzhou Jiangsu China; ^3^ Department of Histology and Embryology, School of Basic Medical Sciences Nanjing Medical University Nanjing China; ^4^ Reproductive Medicine Center The Fourth Affiliated Hospital of Jiangsu University Zhenjiang China; ^5^ Institute of Reproductive Sciences, Jiangsu University Zhenjiang China

**Keywords:** E3 ubiquitin ligase, ovarian cancer, TRIM8, VDAC2

## Abstract

**Background:**

Ovarian cancer is a common gynecological tumor with high malignant potential and poor prognosis. TRIM8, is involved in the development of various tumors, but its precise regulatory role in ovarian cancer is still unknown.

**Aims:**

The aim of this study was to explore the specific mechanism by which TRIM8 regulates ovarian cancer.

**Materials and Methods:**

We used bioinformatics analysis to screen for high expression of TRIM8 in ovarian cancer. The expression of TRIM8 in healthy and cancerous ovarian tissues was assessed by immunofluorescence. TRIM8 was silenced or overexpressed in ovarian cancer cell lines, with cell proliferation and migration evaluated by CCK8, transwell and clonal formation assays. The effect of TRIM8 on ovarian cancer cells in vivo was assessed by subcutaneous tumor formation experiments in nude mice. The potential interacting protein VDAC2 was identified by mass spectrometry. The mechanism underlying TRIM8 regulation of VDAC2 was evaluated by co‐immunoprecipitation and western blotting.

**Results:**

TRIM8 was overexpressed in ovarian cancer. TRIM8 promoted the proliferation and migration of ovarian cancer cells in vitro and the growth of subcutaneous tumors in mice in vivo. TRIM8 interacted with VDAC2, weakened the stability of the protein, and promoted its polyubiquitination and subsequent degradation. Knockdown of VDAC2 increased the resistance of ovarian cancer cells to iron death, whereas overexpression of VDAC2 attenuated ovarian cancer progression induced by TRIM8 overexpression.

**Discussion:**

TRIM8 promotes ovarian cancer proliferation and migration by targeting VDAC2 for ubiquitination and degradation, these finding may provide new targets for the treatment of ovarian cancer.

**Conclusion:**

TRIM8 degraded VDAC2 through the ubiquitination pathway, increased the resistance of ovarian cancer cells to iron death, and promoted the proliferation and migration of ovarian cancer.

## Graphical abstract

TRIM8 degraded VDAC2 through the ubiquitination pathway, increased the resistance of ovarian cancer cells to iron death, and promoted the proliferation and migration of ovarian cancer. This study is the initial demonstration of the cancer‐promoting effects of TRIM8 in ovarian cancer by means of a distinct mechanism that involves the regulation of VDAC2.

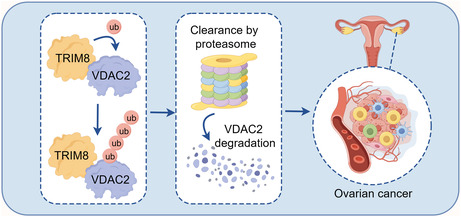

## 1 INTRODUCTION

Ovarian cancer (OC) is a prevalent form of gynecological malignancy, characterized by the highest fatality rate among all cancers specific to females and generally presenting a bleak prognosis.^1^ The main treatment methods for OC include surgical intervention, chemotherapy and targeted therapy,^2^ with the latter mainly involving PARP inhibitors and anti‐angiogenesis drugs.^3^, ^4^ Exploration of the molecular mechanism underlying OC tumorigenesis may identify additional therapeutic targets.

Protein ubiquitination is a common post‐translational modification.^5^, ^6^ The ubiquitin‐mediated degradation of oncogene products and/or tumor suppressor factors has been associated with tumor development,^7^ with the dysregulation of ubiquitination and de‐ubiquitination observed in many types of cancer.^8^ Ubiquitination is an ATP‐dependent cascade process, in which ubiquitin activator E1 binds to and activates ubiquitin, subsequently transferring activated ubiquitin to the ubiquitin coupling enzyme E2, with ubiquitin ligase E3 transferring ubiquitin from E2 to the substrate.^9^, ^10^ In the ubiquitin‐proteasome system, E3 ubiquitin ligase plays a crucial role in determining the specific recognition of target proteins.^11^

The family of tripartite motif (TRIM) proteins, alternatively referred to as RING, B‐box, and coiled‐coil (RBCC) proteins, possess a distinctive structural composition. These proteins consist of an N‐terminal TRIM region that encompasses three zinc‐binding domains, a RING (R) domain, one or two B‐boxes (B1 and B2), and a coiled‐coil region.^12^ TRIM proteins rely on the RING domain to become E3 ubiquitin ligases. After binding to the substrate, the target protein is labeled by ubiquitin and destroyed.^13^, ^14^ Alterations in TRIM protein expression have been found to change the ubiquitination levels of various target proteins, leading to tumorigenesis.^15^ For example, TRIM15,^16^ TRIM59,^17^ and TRIM44,^18^ along with other TRIM proteins, were reported to be involved in the development of pancreatic cancer, osteosarcoma, and esophageal cancer, respectively.

The TRIM8 gene is located on chromosome 10q24.3; its transcription yields an mRNA of about 3.0 kb, which is translated into a protein with a molecular weight of 61.5 kDa.^19^ In addition to acting as an oncogene by favorably regulating the NF‐kB pathway, TRIM8 has been demonstrated to play a significant role in regulating the tumor inhibitory activity of P53.^20^ To date, however, the pathological and clinical roles of TRIM8 in OC have not been determined.

The present study evaluated the role of TRIM8 in OC by analyzing the difference in its expression in OC and normal ovarian tissue samples, as well as determining whether it promotes the proliferation and migration of OC cells. In addition, this study evaluated the interaction between TRIM8 and VDAC2 and explored the associated ubiquitination mechanisms.

## 2 MATERIALS AND METHODS

### 2.1 Dataset analyses

The scRNA‐seq dataset GSE184880, consisting of five normal ovarian tissue samples and seven OC samples, was downloaded from the GEO database. This dataset was analyzed using the Seurat package in R, retaining cells containing ≤20% of mitochondrial genes and filtering out cells with ≤300 or ≥5000 genes (nFeature RNA). The data were normalized using the LogNormalize function, cell clusters were labeled using the SingleR package, and the CellChat software package was used for cell–cell communication correlation analysis. In addition, 356 OC patients were selected from the TCGA database and Kaplan–Meier (KM) analyses were performed to evaluate the relationship between the expression of key genes and relapse‐free survival (RFS).

### 2.2 Cell culture

The OC cell lines OVCAR3 and A2780 were purchased from Meisen CTCC (Hangzhou, Zhejiang, China). OVCAR3 cells were maintained in RPMI‐1640 medium (Gibco, USA) supplemented with 20% fetal bovine serum (FBS; Excell Bio, New Zealand) and 1% penicillin–streptomycin (NCM, China) at a temperature of 37°C in a CO_2_ incubator with 5% CO_2_. On the other hand, A2780 cells were cultured in RPMI‐1640 medium supplemented with 10% FBS.

### 2.3 Cell transfection

The plasmids pcDNA3.1‐Flag‐TRIM8, pEGFP‐VDAC2, and pRK5‐HA‐Ub, overexpressing human TRIM8, VDAC2, and Ub‐WT, respectively, were constructed by PCR amplification of each cDNA and cloning into the pcDNA3.0 vector (Invitrogen). All siRNAs used in this study were obtained from Beijing Tsingke Biotech and transfected into cells. Erastin, an inducer of iron death, was obtained from MedChemExpress, (HY‐15763), Shanghai, China. This medication was administered to the transfected cells for 24 h at a concentration of 5 μM in the working fluid.

The siRNAs used in this study included: NC siRNA (5′‐UUCUCCGAACGUGUCACGU‐3′); TRIM8 siRNA 1# (5′‐GGAGAUCCGAAGGAAUGAA‐3′); TRIM8 siRNA 2# (5′‐GCUGCCGUGCAAACACAACUU‐3′); VDAC2 siRNA 1# (5′‐GAUACUACCUUCUCACCAA‐3′); and VDAC2 siRNA 2# (5′‐GAUCUUGACACUUCAGUAA‐3′).

### 2.4 Cell proliferation assays

The transfected cells, at a density of 2500 cells per well, were transferred to 96‐well plates. To each well was added 20 μL CCK‐8 solution (Beyotime Institute of Biotechnology, Nantong, Jiangsu, China) at 0, 24, 48, 72, and 96 h after cell adhesion. After a duration of 4 h, the microplate reader (Bio‐Rad Model 680, Richmond, CA, USA) was employed to measure the absorbance at 450 nm for each well, following the established protocol.^21^, ^22^ In addition, colony formation tests were performed to evaluate cell proliferation capacity. Cells, at a density of 1000 cells per well, were transferred to six‐well plates and cultured for 2 weeks. On the designated day of collection, the colonies were treated with methanol for a duration of 15 min to fix them. Subsequently, they were stained with a solution of 0.1% crystal violet (Beyotime) for a period of 30 min. The colonies were then observed and quantified using a bright‐field microscope (Carl Zeiss, Oberkochen, Germany), following a previously established protocol.^23^, ^24^

### 2.5 Cell migration assays

Cell migration was evaluated by transwell assays. Briefly, 48 h following transfection, the cells were introduced into each upper chamber with a cell density of 50,000 cells per unit volume. After a 48‐h incubation period, the chambers were fixed and stained according to the previously mentioned protocol for the purpose of colony collection.

### 2.6 In vivo tumor growth assay

To determine whether TRIM8 affects the proliferation of OC cells in vivo, its effects on subcutaneous tumor formation in nude mice were evaluated. We raised ten 7‐week‐old female BALB/c nude mice in a controlled environment free of any specific pathogens. ShRNAs were packaged in lentivirus vectors and obtained from Beijing Tsingke Biotech. Transfection of shRNA was performed with Lipo2000 (Invitrogen, Carlsbad, USA) according to the manufacturer's instructions, following a previously established protocol.^25^, ^26^, ^27^ OVCAR3 cells, which had been genetically modified with short hairpin (sh)‐TRIM8 or a sh‐negative control (NC), were rinsed twice with phosphate‐buffered saline (PBS) and then treated with trypsin for digestion. Finally, the cells were resuspended in PBS at 3 × 10^7^ cells/mL. A volume of 200 μL of cells in suspension was subcutaneously injected into each nude mouse, with cells transfected with sh‐TRIM8 and sh‐NC being injected on opposite sides. The size of the tumors (0.5 × length × width^2^) was assessed at 3‐day intervals. Following a period of 12 days, the mice were euthanized, and the subcutaneous tumors were excised, weighed, and visually documented. The Ethics Committee of Nanjing Medical University granted approval for all animal protocols.

### 2.7 Immunofluorescence

Fresh subcutaneous tumor tissues were fixed with 4% paraformaldehyde for 48 h. The samples were resized to resemble soybeans, dehydrated using ethanol, made transparent using xylene, embedded in paraffin, sliced into 6 μm sections, deparaffinized using xylene, and rehydrated using ethanol at varying concentrations. The antigen was prepared in a sodium citrate buffer with a pH of 6.0 at a concentration of 10 mmol/L for a duration of 20 min. The sections were then blocked using a 5% w/v bovine serum albumin (BSA) solution from Sunshine, Nanjing, China, and incubated overnight at 4°C with a primary antibody against Ki67 from Abcam. Following a wash with PBS, the sections were incubated with secondary antibodies from Thermo Scientific and observed using confocal laser microscopy from Zeiss LSM710, Carl Zeiss, Oberkochen, Germany.

The OC and normal tissue microarray were obtained from Zhongke Huaguang Biotech Co., Ltd. (Xi'an, China) and incubated with a primary antibody against TRIM8 from ProteinTech.

### 2.8 RNA extraction and reverse‐transcription quantitative PCR (RT‐qPCR)

Total RNA was extracted from cells with TRIzol (Vazyme) reagent and reverse‐transcribed to cDNA with HiScript III RT Super Mix with a qPCR kit (R323‐01, Vazyme). Real‐time PCR was performed on a 7500 system (Applied Biosystems, Foster City, CA, USA) with SYBR Green Master Mix (Novoprotein Scientific Inc., Shanghai, China) and specific primers for human TRIM8 (forward, 5′‐ATCCTGATGGACAGGACCCA‐3′, and reverse, 5′‐ AGGGGCCTTCTAGCATTTTCC‐3′); human VDAC2 (forward, 5′‐TTGCTGGCTACCAGATGACC‐3′, and reverse, 5′‐ ACCTGATGTCCAAGCAAGGT‐3′); and human 18S rRNA (forward, 5′‐AAACGGCTACCCATCCAAG‐3′, and reverse, 5′‐CCTCCAATGGATCCTCGTTA‐3′).

### 2.9 Liquid chromatography/mass spectrometry (LC/MS) analysis

Flag‐TRIM8 plasmid was transfected into OVACR3 cells, and the cells treated with RIPA lysis buffer 72 h later. Flag‐TRIM8 was precipitated with anti‐Flag magnetic beads. The immunoprecipitation products were detected by LC/MS, as previously described.^28^, ^29^

### 2.10 Western blotting

Cells were harvested at a time interval of 48–72 h following transfection, and the extraction of total proteins was carried out using lysis buffer (RIPA, Beyotime). After centrifugation, the supernatants were collected, and their protein concentrations were quantified using a bicinchoninic acid (Beyotime Biotechnology) kit. Protein samples were denatured by heating at 100°C for 10 min, and 20 μg aliquots of proteins were loaded onto each lane of a sodium dodecyl sulfate–polyacrylamide gel. After the completion of electrophoresis, the proteins were transferred onto polyvinylidene fluoride membranes. These membranes were subsequently blocked with a solution containing 5% nonfat milk at room temperature for a duration of 1 h. Following this blocking step, the membranes were exposed to various primary antibodies and incubated overnight at a temperature of 4°C. Subsequently, the membranes were washed three times using Tris‐buffered saline plus Tween‐20 and then incubated with secondary antibodies conjugated with horseradish peroxidase for a duration of 1 h at room temperature. Finally, the membranes were washed three times again. Images were obtained with an ECL Prime western blotting detection system and analyzed using Image‐Pro Plus software 6.0, as described.^30^, ^31^ The antibodies used included anti‐TRIM8 (1:1000; cat. no. 27463‐1‐AP; ProteinTech), anti‐VDAC2 (1:1000; cat. no. 11663‐1‐AP; ProteinTech), anti‐tubulin (1:3000; cat. no. 11224‐1‐AP; ProteinTech), anti‐HA (1:500; sc‐7392; Santa Cruz Biotechnology), anti‐GFP (1:1000; ab290; Abcam), and antiFlag (1:1000; F9291; Sigma).

### 2.11 Protein half‐life assay

OVCAR3 cells were genetically modified with either non‐targeting control siRNA or TRIM8 siRNA. Subsequently, these cells were treated with cycloheximide (CHX) at a concentration of 100 μg/mL to inhibit protein synthesis. After a period of 48–72 h following transfection, the cells were harvested and the expression of VDAC2 was assessed using western blot analysis.

### 2.12 Co‐immunoprecipitation

Cells collected 48–72 h after transfection were incubated with RIPA lysis buffer (Beyotime) on ice for 1 h. The samples were centrifuged, the supernatants were decanted, and 20 μL Dynabeads Protein A (Invitrogen) were added to each supernatant, followed by incubation at a slow speed for 2 h to remove non‐specific proteins. Subsequently, the lysates were incubated with anti‐IgG antibody (Fcmacs Biotech) or with magnetic beads bearing anti‐Flag antibody (Sigma) or anti‐GFP antibody (Proteintech) at 4°C overnight (12–16 h), with the lysates incubated with anti‐IgG subsequent incubated with Dynabeads Protein A for 3 h. The immunoprecipitates underwent three washes using RIPA lysis buffer, followed by elution using sodium dodecyl sulfate buffer. The eluted samples were then heated at 100°C for a duration of 10 min and subjected to electrophoresis on sodium dodecyl sulfate–polyacrylamide gels.

### 2.13 Ubiquitination assay

Cells were transfected with specific plasmids and incubated for a duration of 72 h. Subsequently, the cells were subjected to a treatment of 20 μM MG132 for a period of 6 h prior to sample collection. The cells were then lysed using RIPA lysis buffer, with the addition of PMSF to prevent protease activity. VDAC2‐GFP was precipitated with anti‐GFP magnetic beads. Subsequently, the cells were incubated with anti‐Flag antibody to detect TRIM8, anti‐GFP antibody to detect VDAC2, and anti‐HA antibody to detect Ub.

### 2.14 Statistical analysis

The experiments were conducted in triplicate, and the results were presented as the mean value with the standard deviation. Student's *t*‐tests were employed to compare data between two groups, while one‐way analysis of variance was used to compare data among multiple groups. Statistical analyses were performed using GraphPad Prism software, with a significance level of *p* < 0.05.

## 3 RESULTS

### 3.1 TRIM8 is highly expressed in OC and is correlated with unfavorable prognosis in OC patients

**FIGURE 1:**
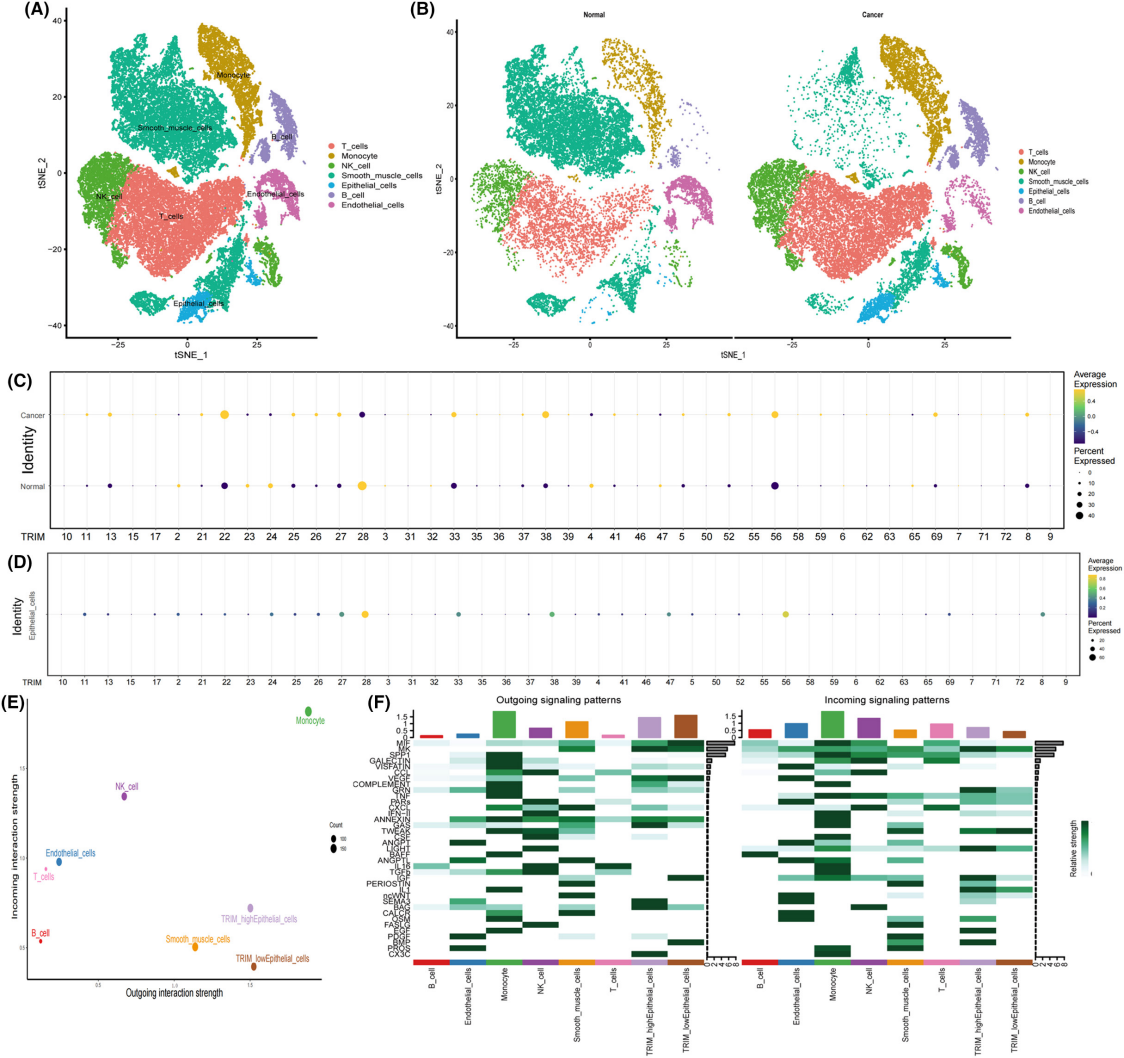
Annotation of cell subpopulations and cell‐chat analysis. (A, B) TSNE plot of cell subpopulations in ovarian cancer (OC) patients. (C) Expression of TRIM proteins in normal ovarian and ovarian cancer tissues. (D) Expression of TRIM proteins in epithelial cells. (E) Communication intensity of various cell subpopulations. (F) Identification of global communication patterns and major signals for specific cell subpopulations.

TRIM8 was identified using a series of bioinformatics analyses. The E3 ubiquitin ligase family in the ubiquitin database–UbiBrowser 2.0 was selected, TRIM proteins were screened out, and the dataset analyzed by scRNA‐seq analysis. Single‐cell data and the distribution of cell subpopulations in normal and cancerous tissues were annotated (Figure 1A,B). The expression of TRIM proteins was evaluated in normal and OC tissues (Figure 1C), and the 10 genes (TRIM13, TRIM22, TRIM27, TRIM25, TRIM26, TRIM33, TRIM38, TRIM56, TRIM69, and TRIM8) with the highest levels of expression in cancer tissues were selected. Because OC is primarily a tumor of epithelial origin, the expression of TRIM proteins was analyzed in epithelial cells (Figure 1D), and the eight most highly expressed genes (TRIM28, TRIM27, TRIM33, TRIM38, TRIM47, TRIM56, TRIM8, and TRIM24) were determined. The two screenings identified five genes in common: TRIM27, TRIM33, TRIM38, TRIM8 and TRIM56. Based on the overall levels of expression of these five genes, epithelial cells were divided into two groups, those with high and low expression, and their involvement in cell–cell communication and signal intensity was determined (Figure 1E,F). Reception signals were found to be stronger in epithelial cells with high than low expression of TRIM proteins.

**FIGURE 2:**
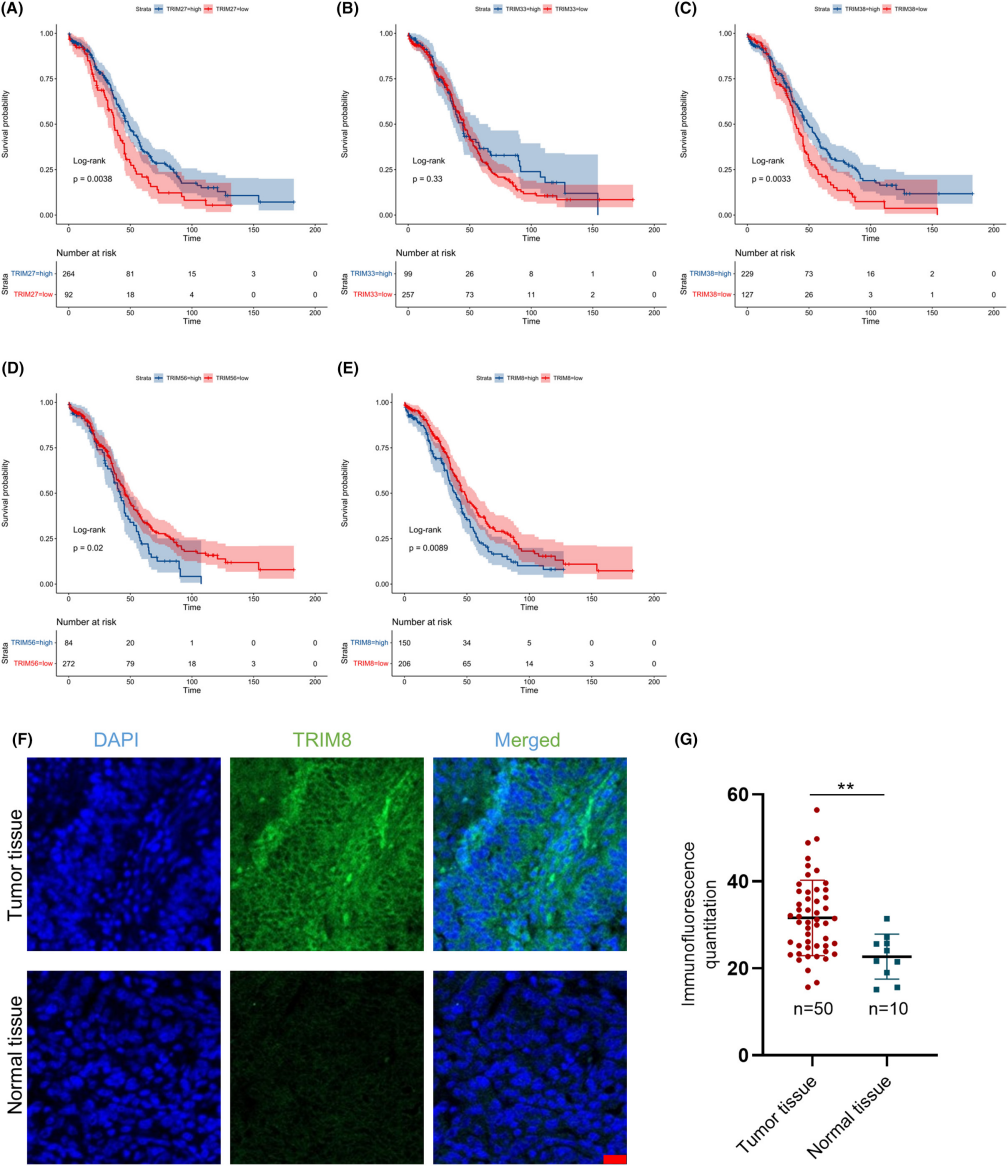
Relationships of key genes with survival in ovarian cancer (OC) patients and validation in tissue samples. (A–E) Kaplan–Meier analyses of the relationships between the expression of key genes and survival in patients with OC. (F) Immunofluorescence detection of TRIM8 protein expression in OC; nuclei were stained with DAPI. Scale bars: 20 μm. (G) Quantification of the intensity of immunofluorescence in (F).

In order to assess the correlation between the screening outcomes and the prognosis of patients diagnosed with OC, a Kaplan–Meier analysis was conducted utilizing prognostic information obtained from the TCGA dataset (Figure 2A–E). Higher levels of TRIM27 and TRIM38 expression were associated with better prognosis in patients with OC, whereas TRIM33 expression levels were not significantly associated with patient prognosis. In contrast, higher levels of expression of TRIM8 and TRIM56 were associated with poorer prognosis in OC patients, with TRIM8 (*p* = 0.0089) being the more significant risk factor for OC. In addition, we purchased OC and normal tissue chips from Zhongke Huaguang Biotech Co., Ltd. (Xi'an, China) to perform immunofluorescence experiments, and the results showed that TRIM8 expression levels were higher in OC than in normal ovarian tissue samples (Figure 2F,G).

### 3.2 TRIM8 promotes the proliferation and migration of OC cells

**FIGURE 3:**
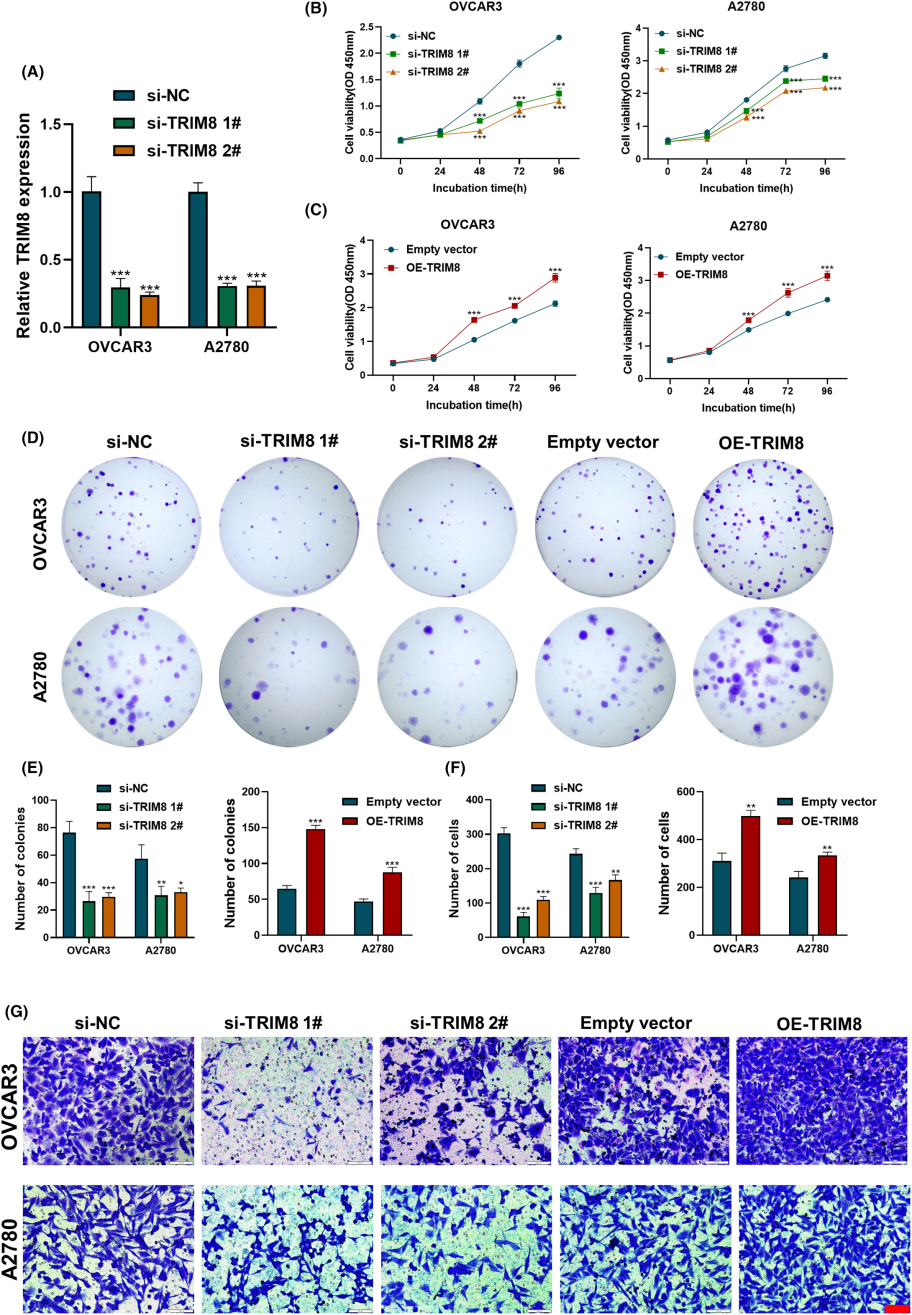
TRIM8 promotes migration and proliferation of ovarian cancer (OC) cells in vitro. (A) TRIM8 mRNA levels after siRNA transfection. (B, C) CCK8 assays showing the proliferation of OC cells after TRIM8 knockdown or otimesverexpression. (D, E) Clone formation experiments, showing that TRIM8 enhances the proliferation of OC cells. (F, G) Transwell experiments showing that the migration of OC cells was reduced after TRIM8 knockdown and enhanced after TRIM8 overexpression. Scale bar, 100 μm. Each experiment was independently repeated three times. *p < 0.05, **p < 0.01, ***p < 0.001 compared with NC or vector alone.

To determine whether TRIM8 affects the proliferation and migration of OC cells, OVCAR3 and A2780 cells were transfected with TRIM8 or NC siRNA, or with a plasmid overexpressing TRIM8 or vector alone. The knockout efficiency of each siRNA was evaluated by RT‐qPCR (Figure 3A). The results of CCK8 proliferation assays demonstrated a notable reduction in the proliferation of OC cells following the suppression of TRIM8 expression. Conversely, overexpression of TRIM8 enhanced the proliferation of OC cells (Figure 3B,C). Clone formation assays showed that the number of colonies was significantly lower following TRIM8 knockdown than after transfection with NC siRNA, whereas the number of colonies was increased in cells overexpressing TRIM8 (Figure 3D,E). The results obtained from transwell assays demonstrated that the downregulation of TRIM8 resulted in a decrease in the migratory capacity of OC cells, while the overexpression of TRIM8 led to an enhancement in OC cell migration (Figure 3F,G). Taken together, assessments of cell phenotype showed that TRIM8 could promote the proliferation and migration of OC cells in vitro.

**FIGURE 4:**
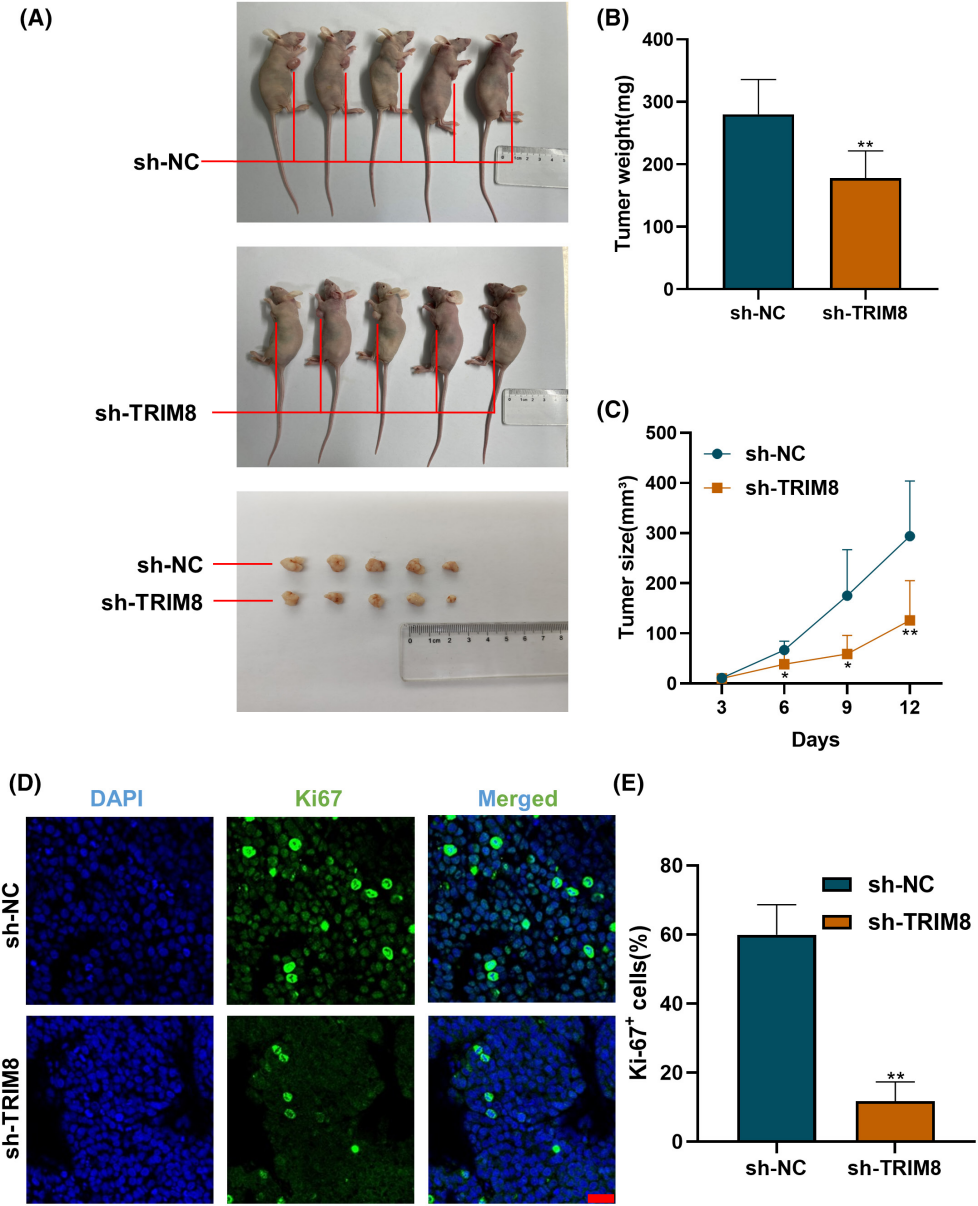
Effects of TRIM8 on ovarian cancer (OC) cell proliferation in vivo. TRIM8‐deficient or control OVACR3 cells were subcutaneously injected into nude mice. (A) Photographs of collected tumors. (B) Measurements of tumor weights. (C) Measurements of tumor volumes every 3 days. (D, E) Immunofluorescence staining of Ki67. Scale bar = 20 μm. *p < 0.05, **p < 0.01.

Due to its robust proliferation capability and high tumor formation rate, the OVCAR3 cell line has been extensively employed in subcutaneous tumor formation experiments in nude mice.^27^, ^32^, ^33^ Consequently, we chose the OVCAR3 cell line for the subcutaneous tumor formation experiments in this study. To determine whether TRIM8 affects OC cell proliferation in vivo, OVCAR3 cells transfected with sh‐TRIM8 and sh‐NC were each injected into the armpits of nude mice, and tumor volumes were calculated every 3 days. After a period of 12 days, the subcutaneous tumors exhibited a notable reduction in size in the group treated with shTRIM8 compared to the control group (Figure 4A–C). Immunofluorescence staining analysis revealed a significant decrease in the number of Ki67 positive cells in the shTRIM8 group (Figure 4D,E), providing additional evidence that TRIM8 plays a role in promoting the proliferation of OC cells in an in vivo setting.

### 3.3 TRIM8 interacts with VDAC2

**FIGURE 5:**
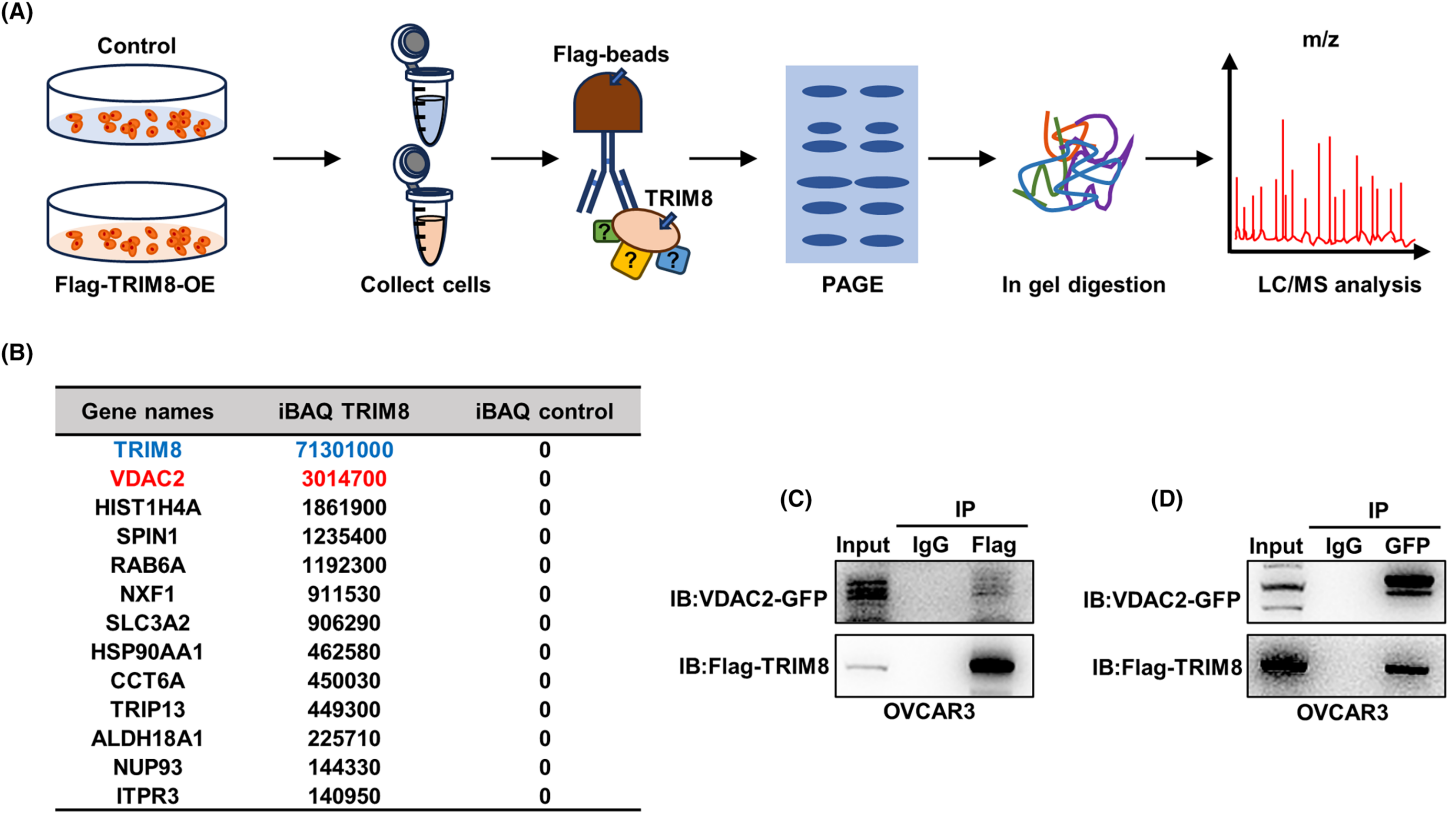
Interactions of TRIM8 with VDAC2. (A) Diagram for the identification of TRIM8‐interacting proteins in OVCAR3 cells. Total protein was extracted from OVCAR3 cells transfected with empty vector or pcDNA3.1‐Flag‐TRIM8, followed by Flag‐bead immunoprecipitation, SDS–PAGE, in‐gel digestion, and LC–MC/MS. (B) Estimation of protein expression levels using the iBAQ algorithm with unique peptide number ≥2. LC‐MC/MS identified 12 proteins that potentially interact with TRIM8. (C, D) Reciprocal immunoprecipitation (IP) assays of OVCAR3 cell lysates with anti‐Flag and anti‐GFP beads.

To evaluate the molecular mechanism by which TRIM8 Promotes OC development, TRIM8 enriched product was extracted by co‐IP and analyzed by mass spectrometry (Figure 5A). Among the molecules found to interact with TRIM8, voltage‐dependent anion‐selective channel 2 (VDAC2) had the highest abundance (Figure 5B). Since the overexpression efficiency of TRIM8 plasmid was higher in OVCAR3 cells (Figure 7B), we used OVCAR3 cells for immunoprecipitation experiments. TRIM8 and VDAC2 were simultaneously overexpressed in OVACR3 cells, and immunoprecipitation experiments were conducted to validate the interaction between TRIM8 and VDAC2 (Figure 5C,D).

### 3.4 Knockdown of VDAC2 can promote proliferation and migration of OC cells under the maintenance of iron death inducer

**FIGURE 6:**
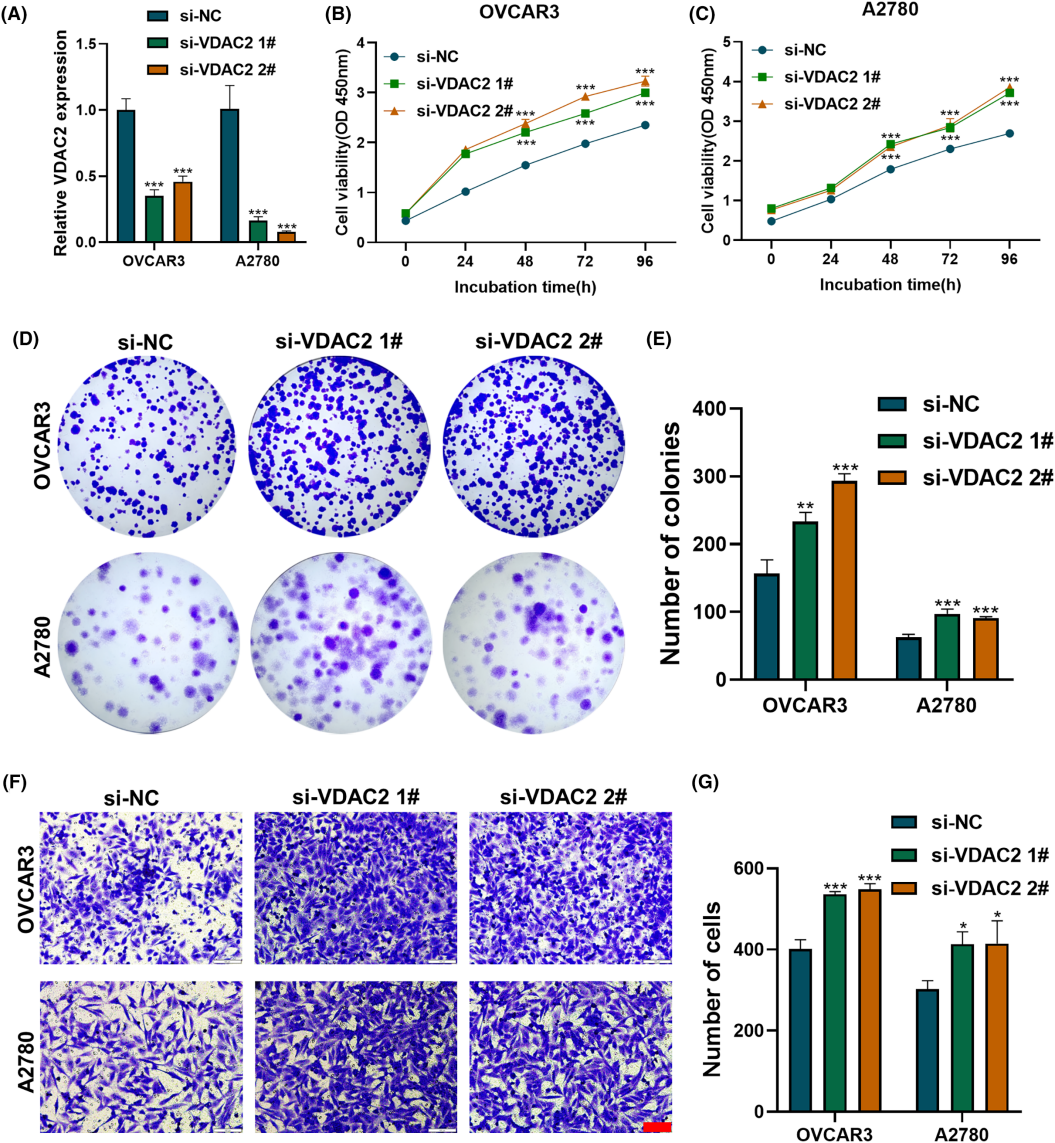
Knockdown of VDAC2 promotes the proliferation and migration of ovarian cancer (OC) cells treated with an iron death inducer, 5 μM erastin, for 24 h. (A) VDAC2 mRNA levels after siRNA transfection. (B, C) CCK8 assays showing that VDAC2 knockdown enhanced the growth of OC cells. (D, E) Colony formation assays, showing that transfection of si‐VDAC2 into OC cells enhanced their proliferation. (F, G) Transwell assays, showing that transfection of si‐VDAC2 enhanced the migration ability of the OC cell lines OVCAR3 and A2780. Scale bar: 100 μm. Each experiment was independently repeated three times. *p < 0.05, **p < 0.01, ***p < 0.001.

In order to investigate the impact of VDAC2 on the proliferation and migration of OC cells, the expression of VDAC2 was suppressed in OVCAR3 and A2780 cells (Figure 6A), while inducing cell death through iron depletion using 5 μM erastin. The assessment of cell viability using CCK8 assay and clonal formation experiments revealed that the knockdown of VDAC2 resulted in an increased proliferation of OC cells (Figure 6B–E). Furthermore, the transwell experiment demonstrated that the suppression of VDAC2 enhanced the migratory capacity of these cells (Figure 6F,G). These experiments indicated that VDAC2 silencing could increase the resistance of OC cells to iron death and that VDAC2 had the potential to inhibit tumor development.

### 3.5 TRIM8 destabilizes VDAC2 through ubiquitination

**FIGURE 7:**
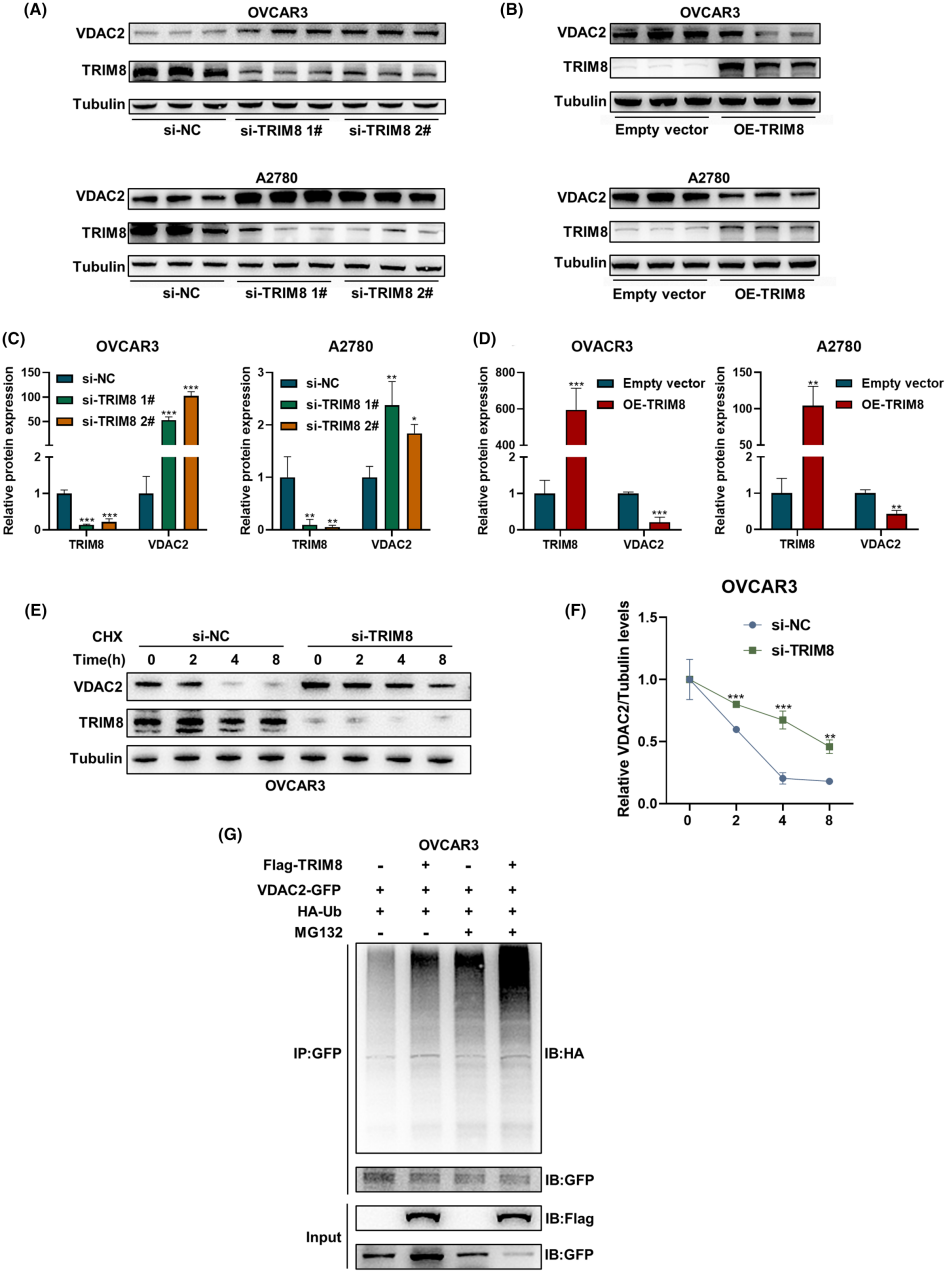
TRIM8 destabilizes VDAC2 through ubiquitination. (A–D) Western blotting assays showing the expression of TRIM8 and VDAC2 proteins in transfected OVCAR3 and A2780 cells. (E, F) Western blotting experiments showing the expression of VDAC2 protein by OVCAR3 cells transfected with TRIM8 siRNA and treated for specified times with cycloheximide (CHX, 100 μg/mL). (G) The plasmids pcDNA3.1‐NC or pcDNA3.1‐Flag‐TRIM8, pRK5‐HA‐Ub, and pEGFP‐VDAC2 were transfected into OVCAR3 cells, followed by incubation in the presence or absence of 20 μM MG132 for 6 h, co‐IP with GFP‐beads, and detection of the ubiquitination level of VDAC2 by western blotting using anti‐HA antibody. Each experiment was independently repeated three times. *p < 0.05, **p < 0.01, ***p < 0.001.

The specific mechanism by which TRIM8 regulates VDAC2 was evaluated by assessing the expression of TRIM8 and VDAC2 in transfected OC cells by western blotting. The expression of VDAC2 was found to be significantly increased after TRIM8 silencing (Figure 7A,C), but decreased after the overexpression of TRIM8 (Figure 7B,D), indicating that TRIM8 could degrade VDAC2 protein. Subsequently, we randomly selected the OVCAR3 cell line to verify the effect of TRIM8 on VDAC2. CHX assays were performed to evaluate the effect of TRIM8 on the half‐life of VDAC2 protein. OVACR3 cells were transfected with si‐TRIM8 and si‐NC and treated with CHX for 0, 2, 4, and 8 h to inhibit protein synthesis, with endogenous VDAC2 protein levels evaluated by western blotting (Figure 7E). The experimental assays conducted in this study demonstrated that the degradation of VDAC2 protein was inhibited and its half‐life was extended following the knockdown of TRIM8 in OVCAR3 cells (Figure 7F). These results provide evidence that TRIM8 plays a role in the degradation of VDAC2 at the protein level, thereby compromising the stability of VDAC2 protein.

Ubiquitination is a common post‐translational modification of proteins and is the main mechanism of protein degradation.^34^, ^35^ TRIM proteins are E3 ubiquitin ligases closely involved in the physiological mechanism of ubiquitination, suggesting that TRIM8 might regulate VDAC2 through ubiquitination. This hypothesis was tested by assessing protein ubiquitination. OVCAR3 cells were transfected with plasmids overexpressing Ub and VDAC2, resulting in TRIM8 overexpression. In addition, the cells were treated with 20 μM MG132 for 6 h to inhibit intracellular proteasome activity. Immunoprecipitation experiments with GFP magnetic beads showed that the ubiquitination level of VDAC2 increased after overexpression of TRIM8. In addition, levels of ubiquitination were increased following MG132 treatment, further demonstrating that TRIM8 regulates VDAC2 through the ubiquitin‐proteasome pathway (Figure 7G).

### 3.6 Overexpression of VDAC2 reversed the phenotype of OC cells affected by overexpression of TRIM8

**FIGURE 8:**
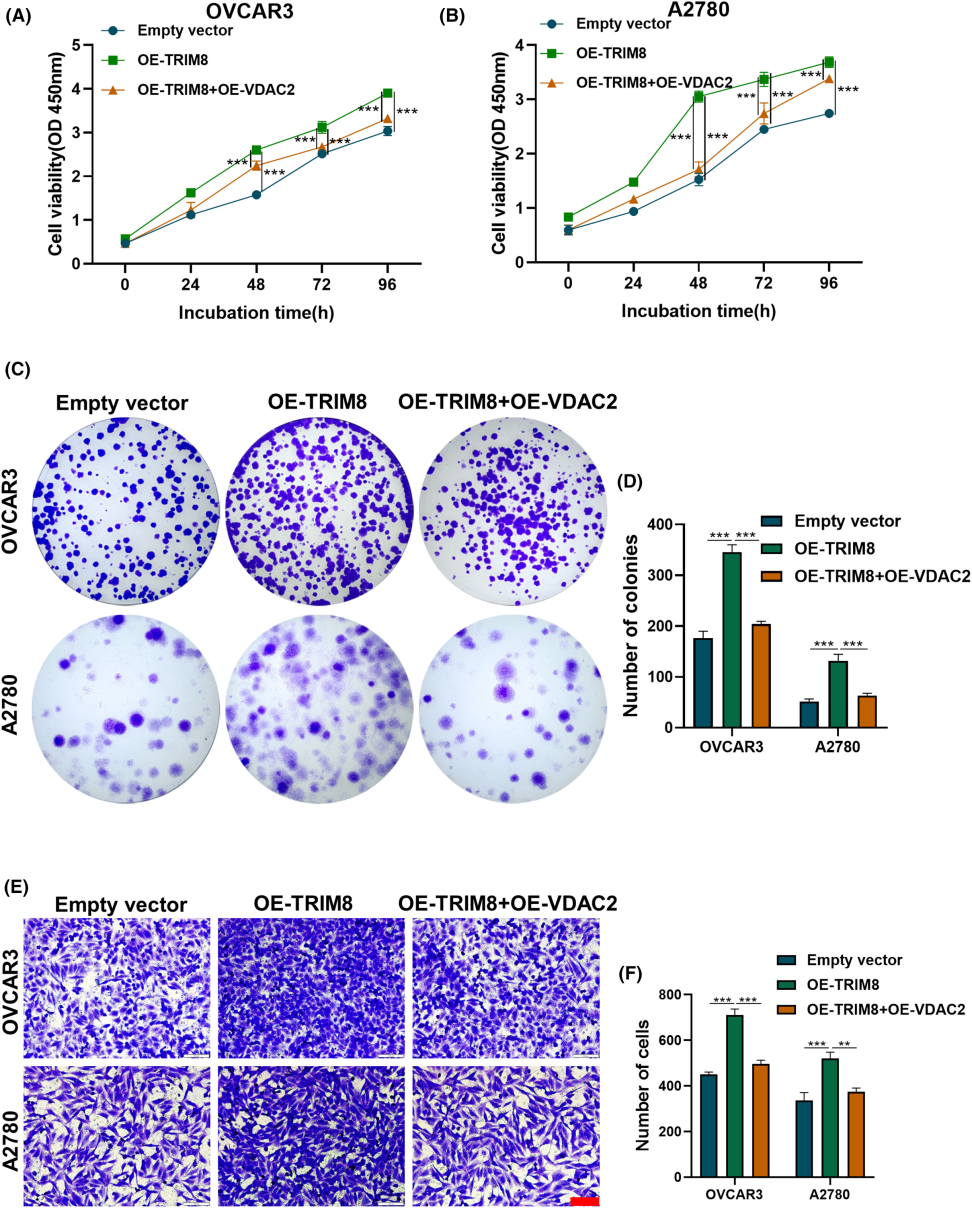
Overexpression of VDAC2 reversed the phenotype of ovarian cancer (OC) cells affected by overexpression of TRIM8. All cells were treated with 5 μM erastin for 24 h. (A, B) CCK8 assays of cell viability. (C, D) Colony formation assays of cell proliferation. (E, F) Transwell assays of cell migration. Scale bar: 100 μm. Each experiment was independently repeated three times. **p < 0.01, ***p < 0.001.

In order to examine the potential role of TRIM8 in promoting OC progression via VDAC2, plasmids containing TRIM8 and VDAC2 were simultaneously introduced into OVCAR3 and A2780 cells. Subsequently, these cells were subjected to treatment with 5 μM erastin, a compound known to induce iron‐dependent cell death. CCK8, transwell, and colony formation assays were subsequently performed to evaluate cell proliferation and migration (Figure 8A–C,E). Overexpression of TRIM8 alone was found to enhance the proliferation and migration of OC cells, whereas overexpression of both TRIM8 and VDAC2 could inhibit these effects (Figure 8D,F). These results suggested that VDAC2 could weaken the OC‐promoting effect of TRIM8, and that TRIM8 could promote OC progression by regulating VDAC2.

## 4 DISCUSSION

Proteins in the TRIM family are characterized structurally by a tripartite motif, consisting of a RING domain (R), one or two B‐boxes (B), and a coil domain (CC).^12^ One of these proteins, TRIM8, has been reported to regulate cell proliferation and participate in the carcinogenesis process.^36^, ^37^ TRIM8 was shown to play a dual role in tumors. Its up‐regulation in renal cell carcinoma was found to restore the tumor inhibitory activity of p53,^38^ but it was also found to promote the survival of Ewing sarcoma by regulating the EWS/FLI oncoproteins.^39^ Bioinformatics analysis in the present study showed that TRIM8 was highly expressed in OC tissues, with this finding verified by immunohistochemistry. Cell function tests demonstrated that TRIM8 could promote the phenotype of OC cells.

Protein ubiquitination is a post‐translational modification and involves the participation of three enzymes: ubiquitin‐activating enzyme (E1), ubiquitin‐conjugating enzyme (E2), and ubiquitin ligase (E3).^40^ The latter protein has been found to select, recruit, and bind specific substrates, suggesting its importance in ubiquitination. E3 ligases can be classified into three distinct families: the RING finger family, the HECT family, and the RBR family.^41^ TRIM8 is a member of the RING finger family. The protein in question does not have a direct binding affinity for ubiquitin. However, it plays a crucial role in facilitating the transfer of ubiquitin molecules from the E2 enzyme that is bound to it, to the specific substrate that it targets.^42^

Using mass spectrometry, the present study identified the protein VDAC2 as interacting directly with TRIM8, with this interaction was verified by immunoprecipitation and western blotting. TRIM8 was found to degrade VDAC2 and reduced the stability of this protein. Overexpression of TRIM8 in OC cells enhanced VDAC2 ubiquitination, indicating that TRIM8 can transfer ubiquitin to VDAC2. Additional studies, however, are required to identify the site on VDAC2 ubiquitinated by TRIM8.

VDACs are a group of specific channel proteins that promote mitochondrial respiration substrates, thereby regulating mitochondrial respiration and energy metabolism.^43^ VDAC2 contributes to oxidative metabolism by participating, along with VDAC1 and VDAC3, in solute transport across the outer mitochondrial membrane (OMM).^44^ In addition, VDAC2 activation plays a key role in iron death, and knockdown of VDAC2 can increase cell resistance to iron death.^45^ Malonylation of VDAC2 may induce mitochondrial dysfunction, increase mitochondrial ROS levels, lead to cell iron death, and inhibit tumor development.^46^ The present study found that VDAC2 knockdown increased the resistance of cells to erastin‐induced iron death, suggesting that VDAC2 silencing could indeed increase cell resistance to iron death, therebt promoting tumor development. In addition, rescue experiments showed that VDAC2 could reduce the cancer‐promoting effect induced by TRIM8. Additional in‐depth studies are required to clarify the specific regulatory mechanisms involving TRMI8, VDAC2 and iron death.

BAX and BAK are members of the BCL‐2 protein family and have essential functions as mediators of intrinsic apoptosis. A previous study found that the interaction with VDAC2 was critical for BAX‐mediated apoptosis, but not BAK. VDAC2 has been shown to promote cell apoptosis and limit tumor development through BAX.^47^ In contrast, another study found that VDAC2 specifically interacted with the inactive conformation of BAK, and cells lacking VDAC2 tended to exhibit enhanced BAK oligomerization and were more prone to apoptosis, so VDAC2 could inhibit cell apoptosis.^48^ Our findings indicated that VDAC2 knockdown increased cell resistance to iron death, suggesting that VDAC2 had the potential to promote apoptosis and subsequently inhibit cancer development. In view of the dual role of VDAC2, more studies are needed in the future to clarify the specific role of VDAC2 in apoptosis and related mechanisms.

**FIGURE 9:**
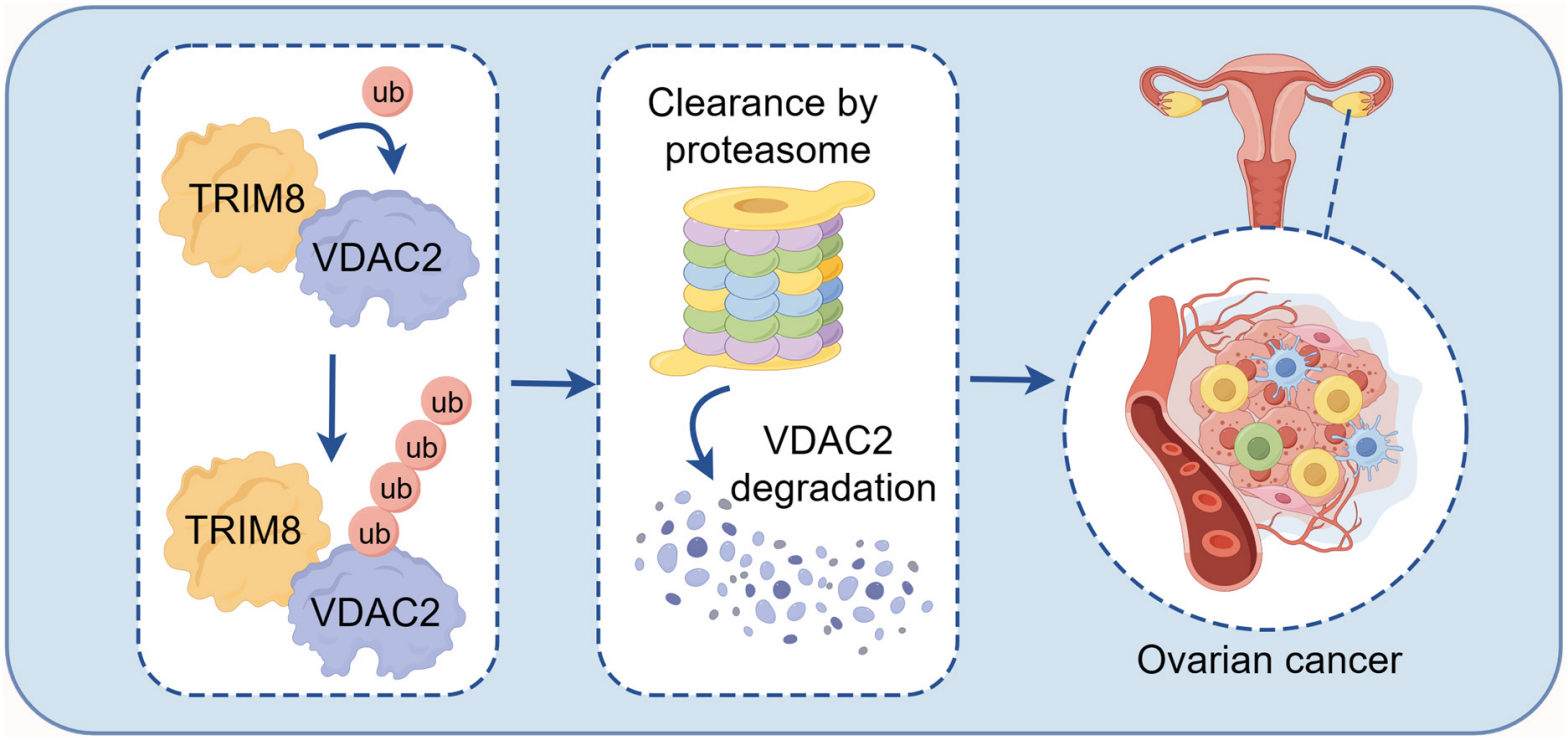
Diagram drawn by Figdraw showing the mechanism by which TRIM8 is involved in ovarian cancer (OC) development.

To our knowledge, this study is the initial demonstration of the cancer‐promoting effects of TRIM8 in OC by means of a distinct mechanism that involves the regulation of VDAC2 (Figure 9). These findings may identify molecular targets for the treatment of OC.

## AUTHOR CONTRIBUTIONS

**Fei Wu:** Data curation (equal); writing – original draft (equal). **Jiaqi Xu:** Data curation (equal); visualization (equal). **Xin Jin:** Writing – original draft (equal); writing – review and editing (equal). **Yue Zhu:** Formal analysis (equal); supervision (equal). **Wenxin Gao:** Data curation (equal); validation (equal). **Meng Liu:** Methodology (equal); validation (equal). **Yan Zhang:** Data curation (equal); resources (equal). **Weifeng Qian:** Funding acquisition (equal). **Xiaoyan Huang:** Formal analysis (equal); visualization (equal). **Dan Zhao:** Writing – original draft (equal); writing – review and editing (equal). **Guannan Feng:** Data curation (equal); resources (equal). **Shunyu Hou:** Funding acquisition (equal); resources (equal). **Xiaoxue Xi:** Data curation (equal); resources (equal); software (equal).

## ACKNOWLEDGEMENTS

We thank all individuals participated in this study.

## FUNDING INFORMATION

This study was funded by Scientific Research Project of Gusu School of Nanjing Medical University (GSKY20210208 and GSKY20210203), Cultivation Special Project of Gusu School of Nanjing Medical University (GSKY20220522), “Science and Education Revitalize Health” Youth Science and Technology Project of Suzhou (KJXW2020028), Special project of diagnosis and treatment technology for key clinical diseases of Suzhou of Jiangsu Province (LCZX202013), Science and Technology Project of Suzhou of Jiangsu Province (SYS2020175), and Key Research Foundation of Zhenjiang Social Development (SH2022029).

## CONFLICT OF INTEREST STATEMENT

The authors declare no conflict of interest.

## ETHICS STATEMENT

All procedures in animal experiments were under the approval of Ethics Committee of Nanjing Medical University.

## Data Availability

The datasets used and/or analyzed during the current study are available from the corresponding author upon reasonable request.
